# Supplementary material for: Neural codes of seeing architectural styles
Source: Sci Rep. 2017 Jan 10;7:40201. doi: 10.1038/srep40201 (PMC5223202; doi:10.1038/srep40201)
Supplement: Supplementary Information [file srep40201-s1.pdf]

[Supplementary Information]

Neural codes of seeing architectural styles

Heeyoung Choo[1] , Jack Nasar[2], Bardia Nikrahei [2], & Dirk B. Walther[3]

[1] Beckman Institute for Advanced Science and Technology, University of Illinois at Urbana-Champaign, Urbana, Illinois, 61801, United States
[2] Department of City and Regional Planning, The Ohio State University Columbus, Ohio, 43210, United States
[3] Department of Psychology, University of Toronto, Toronto, Ontario, M5S 3G3, Canada

Supplementary Methods

Regions of interest

High-level visual regions of interest (ROI) were defined functionally using separate localizer scans. Participants saw one to three runs (7 minutes and 12 seconds each) of blocks of images of faces, scenes, objects, and grid-scrambled objects while responding to image repetitions with a button press. Following motion correction, spatial smoothing (4 mm full width at half maximum Gaussian kernel) and normalization to percent signal change, localizer data were analyzed using a general linear model (3dDeconvolve in AFNI). ROIs were defined as contiguous clusters of voxels with significant contrasts ($q < 0.05$; corrected for multiple comparisons using false discovery rate) in the following comparisons: scenes > (faces, objects) for the parahippocampal place are (PPA), retrosplenial cortex (RSC), and the occipital place area (OPA) [1, 2]; faces > (scenes, objects) for the fusiform face area (FFA) [3]; and objects > scrambled objects for the lateral occipital complex (LOC) [4]. The PPA and RSC were successfully identified in all twenty-two participants. We could not find significant clusters corresponding to the OPA in five participants, the FFA in one participant, and the LOC in two participants. Group statistics of ROI-based results was performed only for the participants for whom we could identify the ROIs.

Primary visual cortex (V1) was defined on each participant's original cortical surface map using the automatic cortical parcellation provided by Freesurfer [5]. Surface-defined V1 was registered back to the volumetric brain separately for each hemisphere using AFNI.

Supplementary Table S1. Multi-voxel pattern analysis decoding results of four visual categories from the ROIs. The group-average decoding accuracies were compared with chance (25%) using one-tailed one sample t-test. P-values were adjusted using false discovery rates for multiple comparison correction. For decoding accuracy rates see Fig. 1 in the manuscript.

		Entry-Level Scene Categories		Architectural Styles		Architects		Faces	
ROI	*df*	*t*	*p*	*t*	*p*	*t*	*p*	*t*	*p*
V1	21	3.120	.003	-.813	.787	1.393	.107	4.695	.000
PPA	21	6.349	.000	4.343	.001	3.432	.008	1.375	.275
OPA	16	3.808	.001	3.337	.004	2.808	.019	-1.007	.988
RSC	21	4.343	.000	3.276	.004	1.635	.088	-2.421	.988
LOC	19	3.974	.001	2.625	.013	1.788	.088	-.984	.988
FFA	20	2.533	.010	2.146	.027	.735	.234	-.803	.988

Supplementary Table S2. Univariate decoding results of four visual categories from the ROIs. We conducted a leave-one-run-out (LORO) classification using mean activities separately for each of the ROIs. Blood-oxygen-level-dependent (BOLD) activity was averaged within each ROI and across experimental blocks. We then computed the mean of the block-averaged BOLD activities across eight runs of every subordinate-level category (i.e., Byzantine) separately per visual category (i.e., architectural styles), leaving out one run for testing. Subordinate-level category labels of testing blocks were assigned following the block label showing the smallest absolute difference (nearest neighbor classifier). We repeated this LORO procedure until each of the nine runs served as a testing run. The group-average decoding accuracy were compared with chance (25%) using one-tailed one sample t-test. P-values were adjusted using false discovery rates for multiple comparison correction. The results showed no successful univariate decoding for any of the four visual categories.

		Entry-Level Scene Categories		Architectural Styles		Architects		Faces	
ROI	*df*	*t*	*p*	*t*	*p*	*t*	*p*	*t*	*p*
V1	21	.709	.918	-1.538	.994	2.060	.077	1.010	.426
PPA	21	-1.314	.918	.476	.643	-.798	.921	.731	.473
OPA	16	-.373	.918	-.610	.994	2.209	.077	-.628	.877
RSC	21	-.825	.918	1.359	.566	.975	.256	.114	.683
LOC	19	-1.446	.918	-2.770	.994	1.870	.077	1.363	.426
FFA	20	-1.067	.918	.471	.643	-1.468	.921	1.826	.959

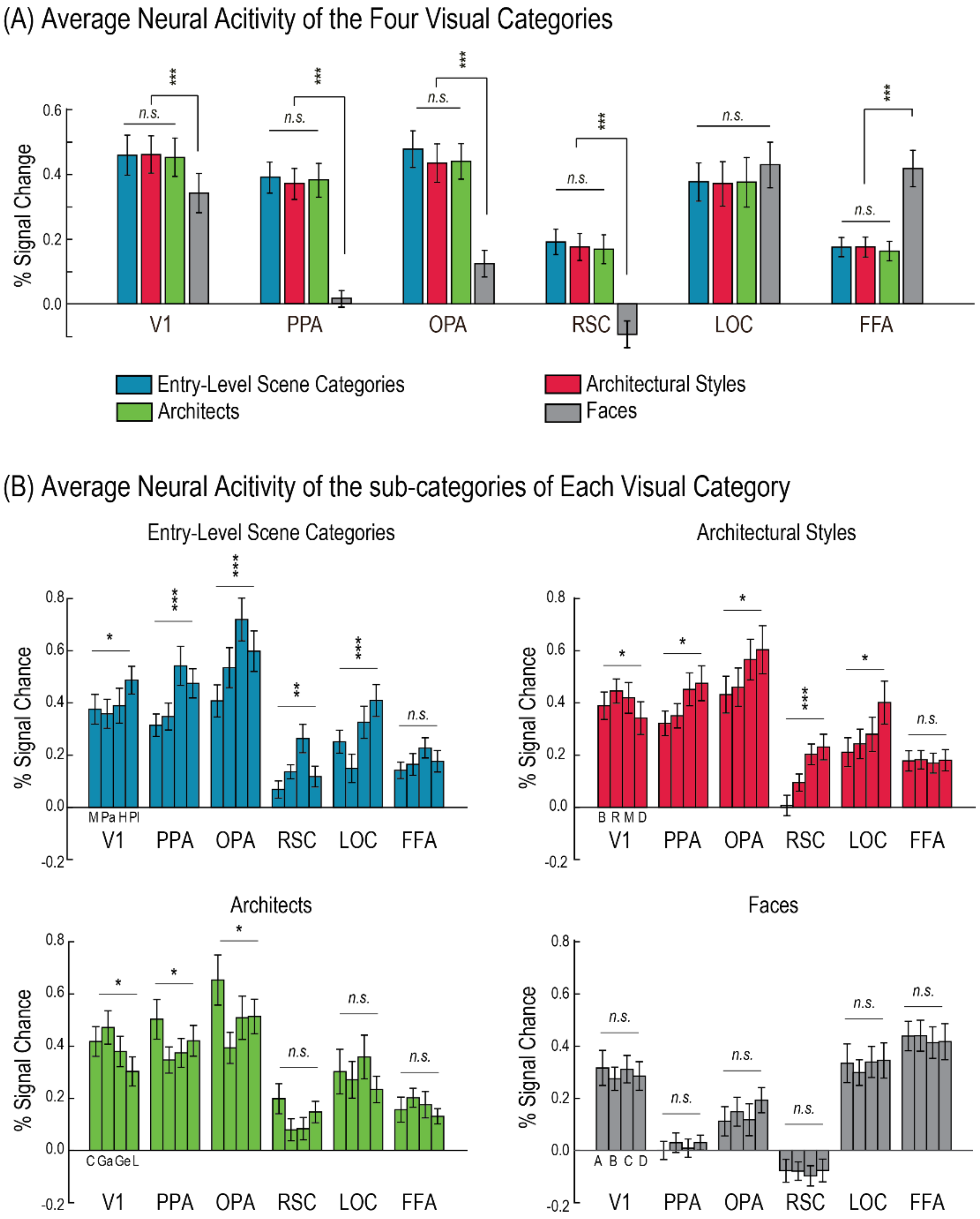

Supplementary Figure 1. The effects of four visual categories and their sub-categories on group average neural activity levels: entry-level scene categories (blue), architectural styles (red), architects (green), and faces (gray). Error bars indicate standard errors of the mean *(SEM)*. (A) As expected, the PPA, OPA, and

RSC showed higher mean activation for scenes, architectural styles, and architects than faces. By contrast, the FFA showed higher activation for faces than for the other visual categories. We found no main effect of visual category in the LOC. A repeated analysis of variance (ANOVA) showed significant main effects of image type in all of the ROIs. The significance of post-hoc tests using the Tukey adjustment is marked above the bars. (B) Average neural activation for subordinate categories: (M)ountains, (Pa)stures, (H)ighways, and (Pl)aygrounds for entry-level scene categories; (B)yzantine, (R)enaissance, (M)odern, and (D)econstructive for architectural styles; Le (C)orbusier, (Ga)udi, (Ge)hry, and (L)loyde-Wright for architects; and (A, B, C, and D) for faces of non-famous individuals. We tested whether the four types of visual categories elicited different levels of mean activity in each of the ROIs. We conducted a mixed-effects analysis of variances (ANOVA) for each ROI separately, using participant group (Architecture vs. Psychology and Neuroscience students) as a between-subjects factor, and visual category (entry-level scene categories vs. architectural styles vs. architects vs. faces) as within-subjects factors. There was no main effect for group nor an interaction between group and visual category. We therefore collapsed the data for the two groups for all further analysis. Differences in mean activity among the subordinate categories for each of the four visual categories were evaluated with one-way ANOVAs. The significance of the subordinate category effect is marked above each bar graph. Despite significant differences by subordinate categories found in subsets of the ROIs, these differences are insufficient for reliably decoding categories based on mean activity levels (see Table S2). *$p < .05$, **$p < .01$, ***$p < .001$

Searchlight analysis

We explored representations of image categories outside of the pre-defined ROIs with a searchlight analysis using the Searchmight Toolbox [6]. The size of the searchlight region was chosen as a 5x5x5 =125 voxel cube to approximate the average size of a unilateral PPA of the participants (159 voxels). The searchlight was centered on each voxel in turn [7], and decoding analysis with leave-one-run-out cross-validation was performed using the voxels within the searchlight regions. Decoding accuracies for the searchlight locations were recorded in a brain map, thresholded at $p < 0.01$ (one-tailed analytical p value), and corrected for multiple comparisons at the cluster level with a minimum cluster size determined separately for each participant, ranging from 4 to 8 voxels ($M = 4.8$, $SD = 0.9$). We evaluated the agreement between the searchlight analysis and the pre-defined ROIs as the fraction of voxels within each ROI that was found to be significantly above chance in the searchlight analysis (Table S3).

For group analysis, anatomical brain volumes of each of the participants were registered to the Montreal Neurological Institute (MNI) 152 template [8]. Searchlight accuracy maps were registered to MNI space using the parameters from the anatomical registration, followed by smoothing with a 2 mm full width at half maximum Gaussian filter. Significance of group-average decoding accuracy versus chance (25%) was assessed with a one-sample, one-tailed t-test ($p < 0.01$), followed by cluster-level correction for multiple comparisons (minimum cluster size of 13 voxels, determined by α probability simulation). See Supplementary Figs. S2-S5 for the resulting group maps.

Supplementary Table S3. Percent of ROI-voxels with significant decoding accuracy in the searchlight analysis. See Figs. S2-S5 for the corresponding searchlight maps. Numbers shown are averages over participants with *SEMs* shown in parentheses. The searchlight map for decoding entry-level scene categories showed the largest amount of overlap with all ROIs. Overlap was smaller for styles, architects and faces. The searchlight map for decoding face identity showed the largest overlap with V1.

ROI	N	Entry-level Scene Categories	Architectural Styles	Architects	Faces
V1	22	13.0 (3.4)	2.9 (0.8)	3.7 (1.4)	9.4 (1.9)
PPA	22	32.9 (5.6)	12.5 (2.8)	7.3 (1.8)	5.8 (2.2)
OPA	17	33.4 (7.6)	15.4 (5.6)	7.7 (2.0)	4.2 (2.2)
RSC	22	20.2 (4.7)	6.6 (2.2)	9.2 (3.1)	5.3 (2.2)
LOC	20	27.1 (4.7)	11.3 (2.7)	8.2 (2.7)	2.2 (0.9)
FFA	21	12.5 (2.1)	6.9 (2.1)	6.4 (1,9)	4.9 (1,4)

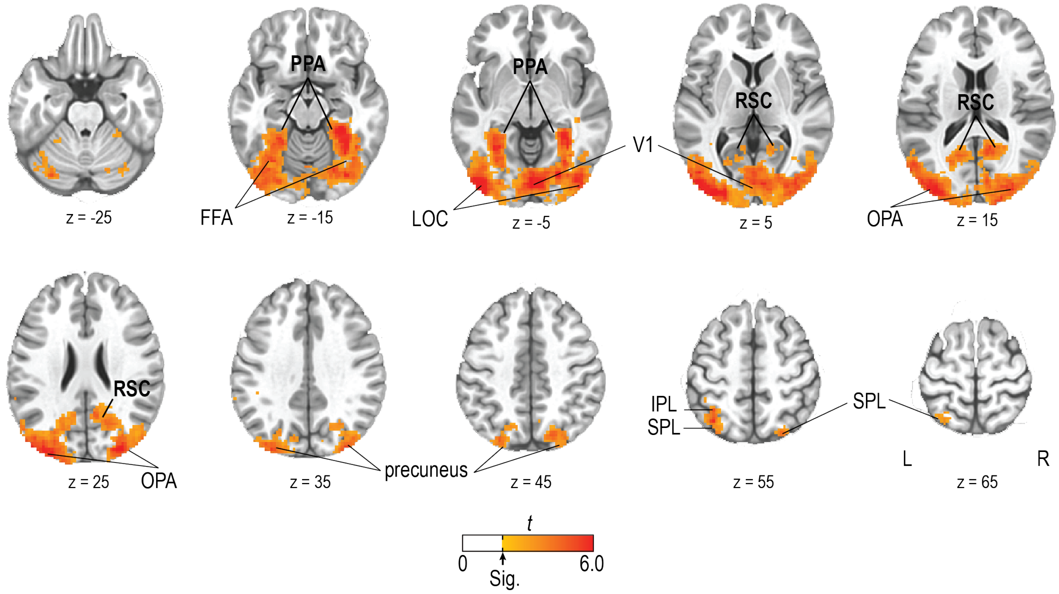

Supplementary Figure S2. Whole-brain searchlight analysis for decoding of entry-level scene categories. The colors indicate t-statistics at searchlight locations. Note that because of the partial coverage of our fMRI scans, anterior temporal and parietal areas and frontal areas were not included in this analysis. Abbreviations: IPL, intraparietal lobule; SPL, superior parietal lobule.

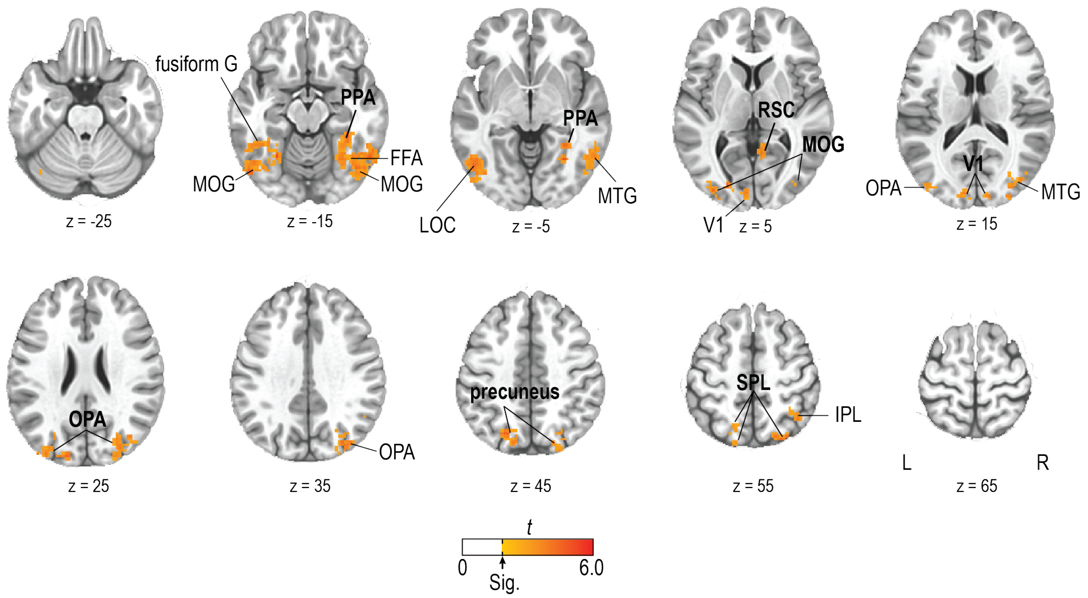

Supplementary Figure S3. Whole-brain searchlight analysis for decoding of architectural styles. The colors indicate t-statistics at searchlight locations. Note that because of the partial coverage of our fMRI scans, anterior temporal and parietal areas and frontal areas were not included in this analysis. Abbreviations: MOG, middle occipital gyrus; MTG, middle temporal gyrus; IPL, intraparietal lobule; SPL, superior parietal lobule.

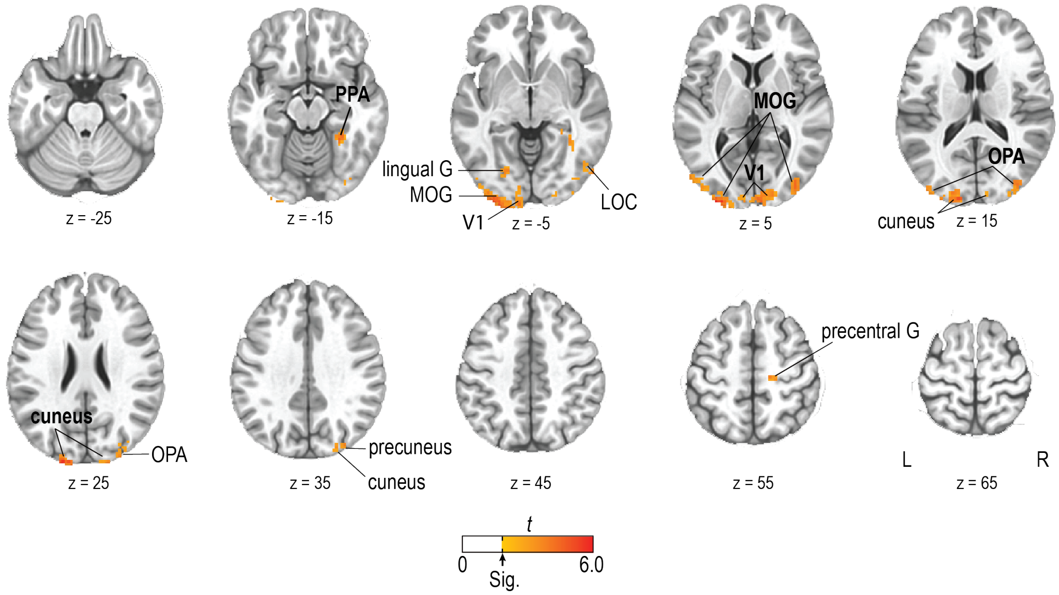

Supplementary Figure S4. Whole-brain searchlight analysis for decoding of architects. The colors indicate t-statistics at searchlight locations. Note that because of the partial coverage of our fMRI scans, anterior temporal and parietal areas and frontal areas were not included in this analysis. Abbreviations: lingual G, lingual gyrus; MOG, middle occipital gyrus; precentral G, precentral gyrus.

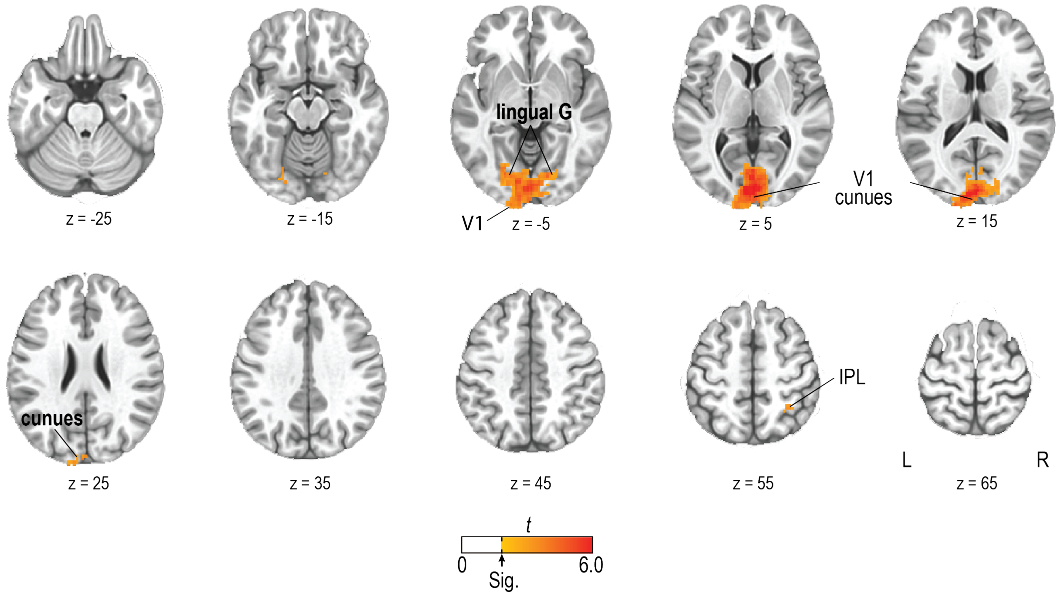

Supplementary Figure S5. Whole-brain searchlight analysis for decoding of facial identities. The colors indicate t-statistics at searchlight locations. Note that because of the partial coverage of our fMRI scans, anterior temporal and parietal areas and frontal areas were not included in this analysis. Abbreviations: lingual G, lingual gyrus; IPL, intraparietal lobule.

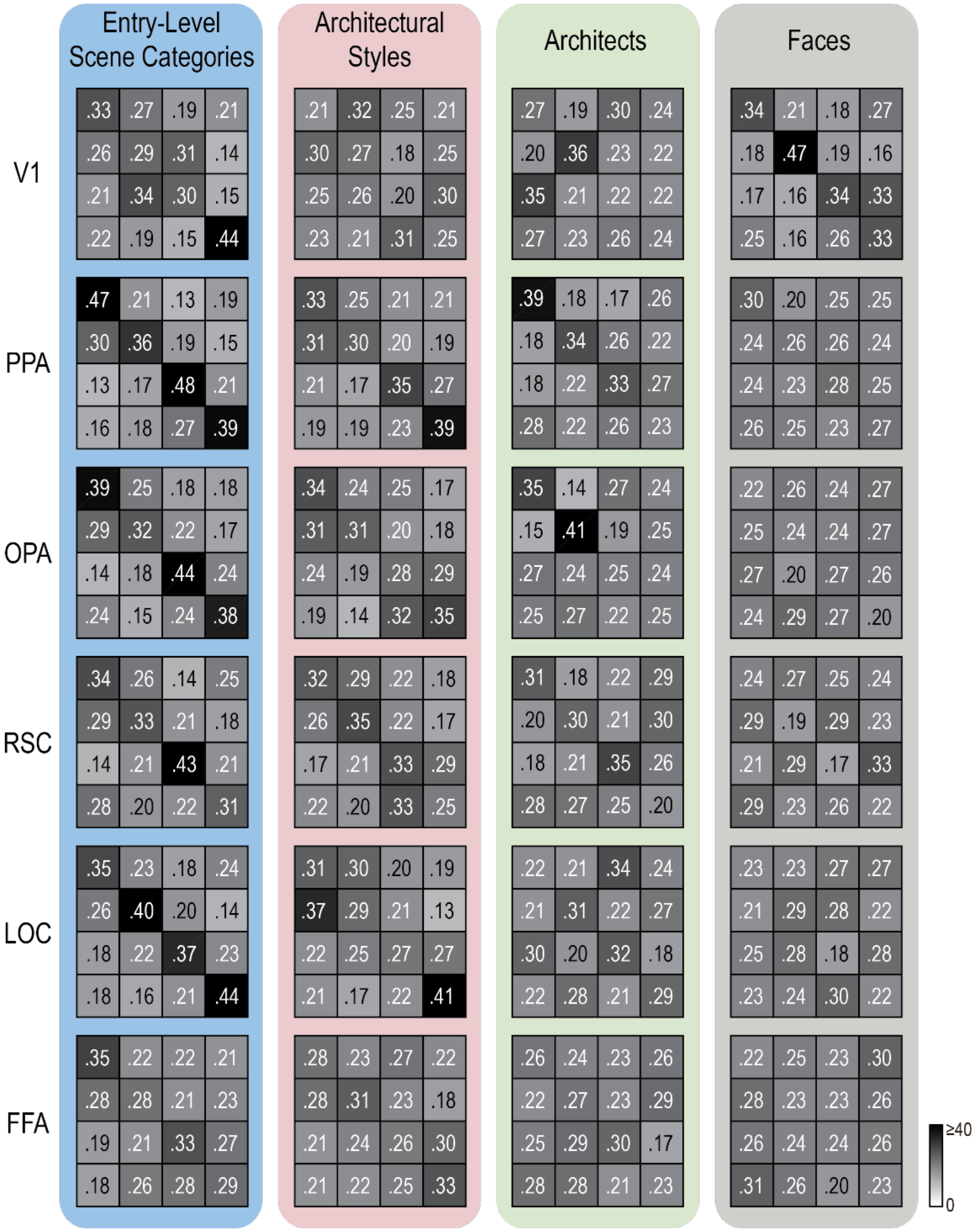

Supplementary Figure S6. Group-average confusion matrices for decoding from the PPA, OPA, RSC, LOC, and FFA for the four visual categories: entry-level scene categories (blue panel), architectural styles (red panel), architects (Green panel), and faces (gray panel). For each confusion matrix, rows (*r*) indicate the ground truth of the presented category, and columns (*c*) represent predictions by the decoder. Individual cells (*r*,*c*) contain the proportion of blocks with category *r*, which were decoded as category *c*. Labels of subordinate categories (from top to bottom and left to right) are: entry-level scene categories: mountains, pastures, highways, and playgrounds; architectural styles: Byzantine, Renaissance, Modern, and Deconstructive; architects: Le Corbusier, Antoni Gaudi, Frank Gehry, and Frank Lloyd-Wright; and faces of four non-famous men, A, B, C, and D.

Supplementary Table S4. Statistical results of a mixed analysis of variance (ANOVA) of decoding accuracy with image type (entry-level scene categories vs. architectural styles vs. architects vs. faces) as a within-subject factor and group (architecture students vs. psychology/neuroscience students) as a between-subject factor, separately for each ROI. The main effect of image type was significant in all six ROIs, suggesting that the amount of decodable categorical information varied significantly across the four image types. However, we found neither a significant effect of group nor a significant interaction between group and visual category in any of the ROIs.

	Group					Visual Category					Group * Visual Category				
	df_1	df_2	F	p	η_p^2	df_1	df_2	F	p	η_p^2	df_1	df_2	F	p	η_p^2
V1	1	20	.666	.424	.032	3	60	7.693	< .001	.278	3	60	.407	.749	.020
PPA	1	20	.087	.772	.004	3	60	8.294	< .001	.293	3	60	1.976	.127	.090
OPA	1	15	.052	.823	.003	3	45	7.198	< .001	.324	3	45	1.612	.200	.097
RSC	1	20	.743	.399	.036	3	60	9.436	< .001	.281	3	60	.289	.833	.014
LOC	1	18	1.562	.227	.080	3	54	7.838	< .001	.254	3	54	.721	.544	.039
FFA	1	19	.250	.623	.013	3	57	3.072	.035	.139	3	57	.728	.540	.037

References

1. Epstein, R. A. & Kanwisher, N. A cortical representation of the local visual environment. *Nature.* **392**, 598-601 (1998).

2. Dilks, D. D., Julian, J. B., Paunov, A. M. & Kanwisher, N. The occipital place area is causally and selectively involved in scene perception. *J. Neurosci.* **33**, 1331-1336 (2013).

3. Kanwisher, N., McDermott, J. & Chun, M. The Fusiform Face Area: A Module in Human Extrastriate Cortex Specialized for the Perception of Faces. *J. Neurosci.* **17**, 4302-4311 (1997).

4. Grill-Spector, K. *et al.* A sequence of object-processing stages revealed by fMRI in the human occipital lobe. *Hum. Brain. Mapp*. **6**, 316-328 (1998).

5. Hinds, O. P. *et al.* Accurate prediction of V1 location from cortical folds in a surface coordinate system. *Neuroimage,* **39**, 1585-1599 (2008).

6. Pereira, F. & Botvinick, M. Information mapping with pattern classifiers: a comparative study. *Neuroimage.* **56**, 476-496 (2011).

7. Kriegeskorte, N., Goebel, R. & Bandettini, P. Information-based functional brain mapping. *PNAS.* **103**, 3863–3868 (2006).

8. Fonov, V. *et al*. Unbiased average age-appropriate atlases for pediatric studies. *Neuroimage*. **54**, 313-327 (2011).
